# The influence of laser-microgrooved implant and abutment surfaces on mean crestal bone levels and peri-implant soft tissue healing: a 3-year longitudinal randomized controlled clinical trial

**DOI:** 10.1186/s40729-021-00382-3

**Published:** 2021-10-04

**Authors:** Assma Syed Ahamed, P. S. G. Prakash, Jasmine Crena, Dhayanand John Victor, Sangeetha Subramanian, Devapriya Appukuttan

**Affiliations:** Department of Periodontics, SRM Dental College and Hospital, Ramapuram, Chennai, India

**Keywords:** Laser-microgrooved implant, Laser-microgrooved prosthetic abutment, Mean crestal bone loss, Peri-implant sulcus depth, Peri-implant biologic width

## Abstract

**Purpose:**

The study aimed to compare clinical and radiographic parameters of peri-implant site of laser-microgrooved implants with either laser-microgrooved or regular machined prosthetic abutment post 3 years of early loading.

**Method:**

Twenty edentulous sites of systemically and periodontally healthy individuals were allocated into two groups in this prospective, two-armed, randomized double-blinded clinical trial in 1:1 ratio, wherein each site received laser-microgrooved implants with either laser-microgrooved or machined prosthetic abutments. Outcome measures included full-mouth plaque (FMPS%) and bleeding score (FMBS%), site-specific plaque (SPS%) and bleeding score (SBS%), peri-implant sulcus depth (PISD mm), and mean crestal bone loss (MCBL mm) evaluated at baseline (6 weeks), 1 and 3 years post-early loading. Mean and standard deviation of all parameters were estimated, between groups and at different time points using independent and paired *t*-test, respectively, Normality was checked using Kolmogorov–Smirnov test and Shapiro–Wilk test, *P* value ≤ 0.05 was considered as statistically significant.

**Results:**

Three-year follow-up of test group showed statistically significant reduction in SPS, SBS, PISD (*P* value ≤ 0.001). The MCBL reduced from 1.93 mm to 0.61 mm (*P* value ≤ 0.001); in other words, a bone gain of 0.15 mm was obtained in the test group.

**Conclusion:**

Within the limitations of the present study, laser-microgrooved implants loaded with laser-microgrooved prosthetic abutments showed superior results clinically and radiographically when compared to loading with machined abutments.

## Introduction

The laudative success of the principle behind ‘Osseointegration’ is being utilized across the world of dentistry in the expanding use of dental implants, to skilfully rehabilitate edentulous spaces. The principle of osseointegration [1] has been shown to provide successful anchorage to root analogues that are fused to the alveolar ridge, providing fixed replacement of missing teeth that fulfill the functional and aesthetic requirements of patients. However crestal bone loss seems to occur as a concurrent phenomenon post-implant placement [2] which has been attributed to the ingress of bacteria to the implant–abutment junction causing peri-implant inflammation which later leads to loss of supporting tissues eventually resulting in peri-implantitis [3]. The initial loss of crestal bone termed as early implant bone loss [4] is a phenomenon defined as the breakdown of the implant–tissue interface beginning at the crestal region in successfully integrated implants that is noted during the first year of prosthetic loading occurring within 1-year post-loading [5]. However, there is lack of unanimity as to the cause of this type of early loss of bone in the loaded implant.

A myriad of research work have been conducted to understand the “Causa Causans” for this observed early implant bone loss. In vitro [6–8] and animal studies [9–12] have demonstrated that surface micro-grooves on the cervical portion of the implant and abutment with a specific shape and depth promote osteoblast and fibroblast proliferation toward the grooves. Thus in order to decrease crestal bone loss around implants, establishing a stable soft tissue seal around the implant abutment interface is necessary in order to ensure prevention of bacterial colonization, and thereby decreasing crestal bone loss [13].

Laser-microgrooved implants have been documented in the recent years [14–17] with improved efficacy in relation to connective tissue attachment and prevention of crestal bone resorption. The concept suggested was that, a direct fibro-collagenous attachment to the implant–abutment complex ensured a direct connective tissue attachment to the altered abutment surface which showed prevention of premature crestal bone resorption [18]. Further laser peening, resulted in grooving of the implant and the abutment surfaces, thus favoring the physical attachment of connective tissue to the grooves hence forming a fibrous capsule arrangement perpendicular to the implant [9]. Studies on laser-microgrooved implants have been very negligible and thus the need for an extensive research on the same is a mandate to expand the research in implant dentistry.

In this study, we try to compare the efficacy of laser-microgrooved implants and laser-microgrooved abutments with laser-microgrooved implants and machined abutments in terms of peri-implant soft tissue and hard tissue healing. To the best of our knowledge this is the first study to compare the laser-microgrooved implants with two different types of abutment connections with a 3-year follow-up.

## Materials and methods

### Ethical committee approval

The study proposal was approved by the Institutional Scientific and Ethical Review Board (SRMU/M&HS/SRMDC/2013/M.D.S-PGSTUDENT/503) and in accordance with the Helsinki Declaration as revised in 2013.

### Study design

**Fig. 1:**
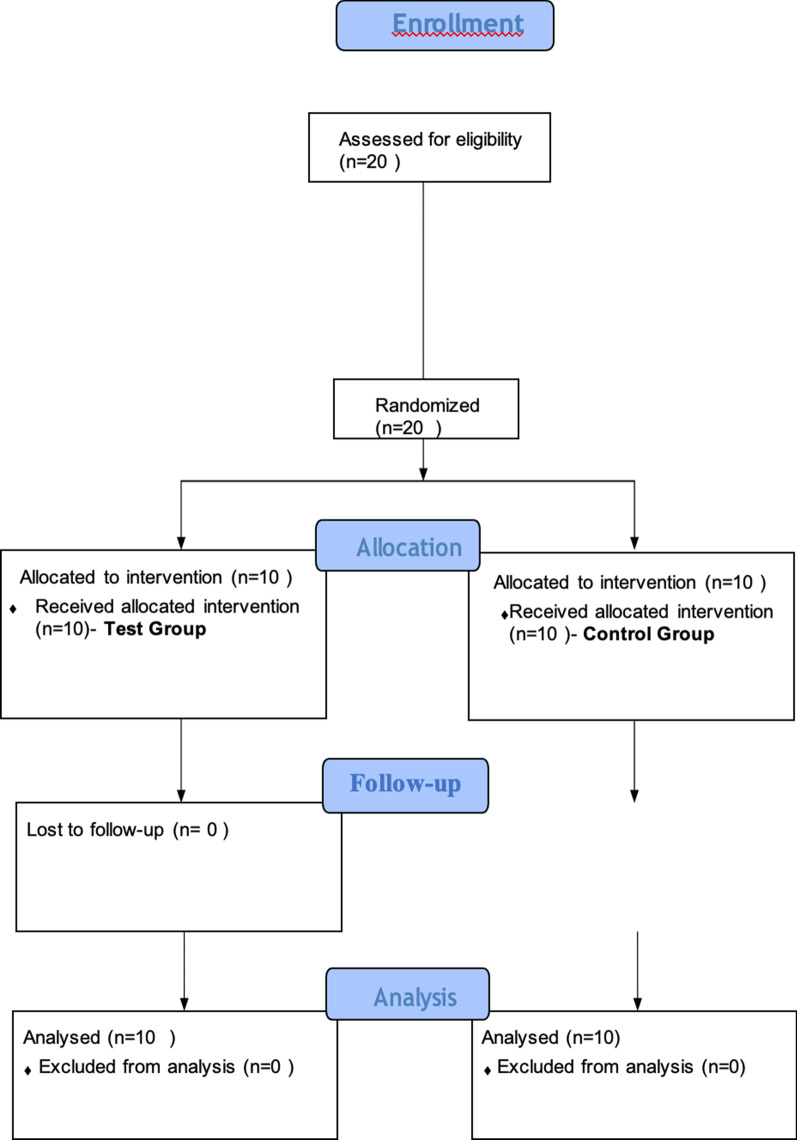
Patient enrollment flowchart

The overall study design was a longitudinal study with a prospective two-armed, parallel group, randomized, double-blinded controlled clinical trial evaluation and has been reported based on the CONSORT statement. The patient allocation is represented as a flowchart (Fig. 1).

### Primary outcome

The primary outcomes included site-specific plaque score (SPS), site-specific sulcular bleeding score (SBS), peri-implant sulcus depth (PISD) measured from the gingival margin to the base of the sulcus, radiographic parameter—mean crestal bone loss measured from the level of the implant abutment junction to the crest of the alveolar bone. The mean crestal bone loss was evaluated using digital radiographic assessment involving the employment of photo-stimulable phosphor plates (PSP), an X-ray positioning device and holding device. The X-ray positioning and holding device was provided with a bite plane where the patient bites at centric relationship and was recorded, with a poly-vinyl siloxane material (heavy body impression material). A guiding arm attached to the X-ray positioning and holding device facilitated reproduction of the position of the tube head, with the same horizontal and vertical angulations, thereby standardizing the radiographic image. The PSP plates were mounted on the X-ray positioning and holding device and radiographs were obtained. Later the PSP were scanned using DIGORA software (DIGORA—the SOREDEX DIGORA is a system that scans imaging plates automatically, with optimized images and displayed on the screen in seconds.) and digital images were obtained. Digital grids were superimposed on the radiographic image using the SOPRO (SOPRO—is a digital imaging software used for creating database of the image). Initially a horizontal line was created to overlap the implant abutment junction after which, a vertical line perpendicular to the implant abutment junction line, passing at the center of the apex of the implant at the mid-axis level was generated. Two parallel lines to the mid-axis line were then produced—one from the mesial aspect and the other from the distal aspect of the implant. Both these lines were spaced 0.1 mm away from the crest module on the implant abutment junction line to the crest of the alveolar bone. The dimensions of these lines were used to measure the crestal bone loss immediately after restoration (baseline), at 1 year and 3 years post-restoration [3].

### Secondary outcome measures

Full-mouth plaque score (FMPS), [19] full-mouth bleeding score (FMBS) [20].

### Sampling

The study population included patients who reported to the out-patient unit of Department of Periodontology, SRM Dental College from February 2014 to February 2017 and who were willing to participate in the study with a signed informed consent. All the willing participants were fully explained about the nature of the surgical protocol and the possible surgical complications.

### Sample size calculation

The sample size calculation for this pilot study was done based on proportionate methodologies with type II beta error set at 85% and type I alpha error at 15%. The resulting estimated sample size set was 10 in each group. Accordingly, 20 edentulous sites were included in the present randomized controlled clinical trial.

### Randomization

**Fig. 2:**
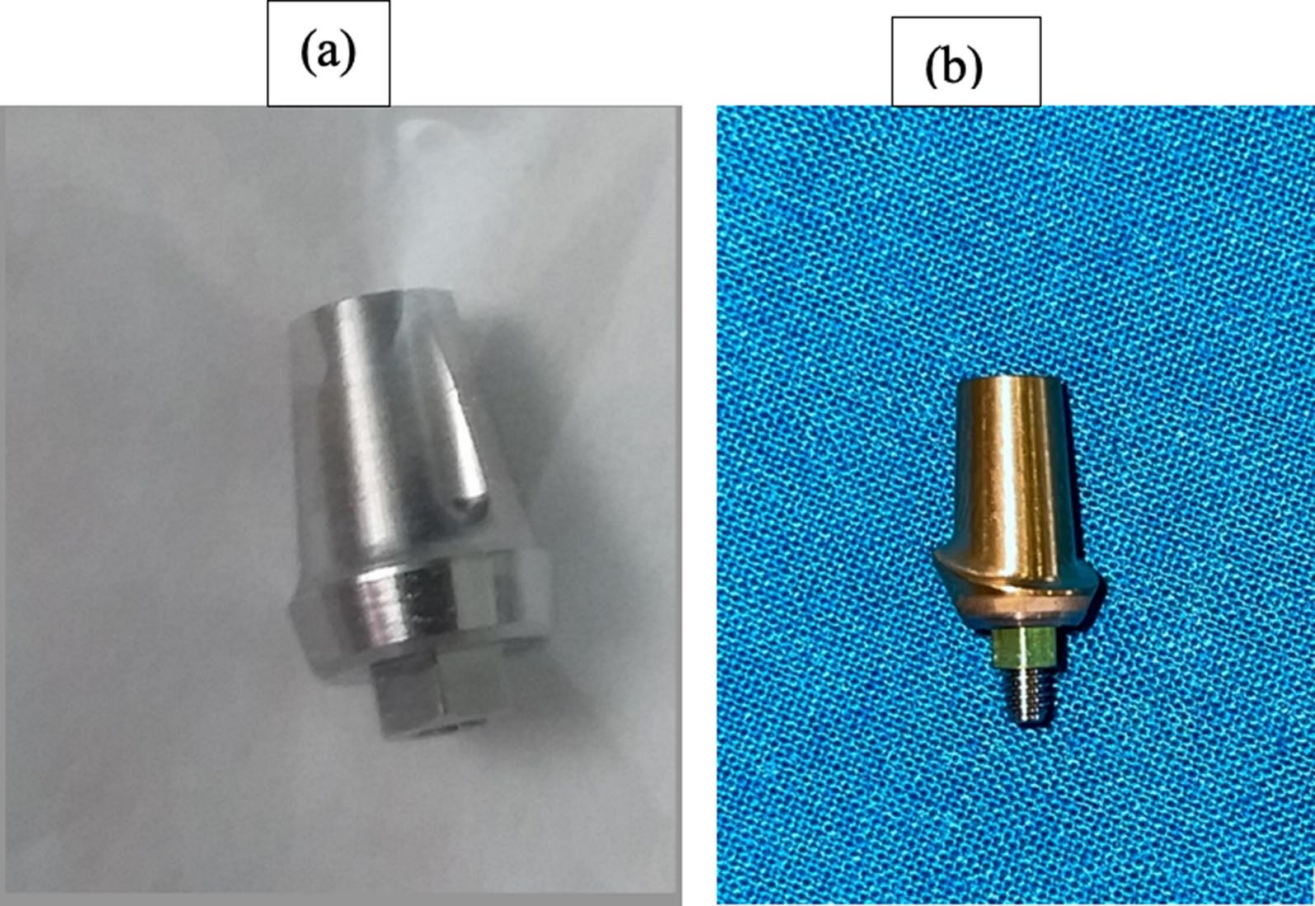
Prosthetic abutments used for loading. **a** Regular Machined abutments, **b** Laser microgrooved abutments

The sample size of 20 partially edentulous sites was divided into two groups. The allocation of the treatment for each patient was done on a random basis with 1:1 allocation ratio, with edentulous sites being serially recruited and assigned with numbers according to their enrollment in the study. The odd numbers were considered as the control group with patients receiving laser-microgrooved implants followed by loading with regular machined abutments and the even numbers were considered as the test group with patients receiving laser-microgrooved implants followed by loading with laser-microgrooved abutments (Fig. 2a, b). All the implants were placed by a single calibrated surgeon who after placing the implants was given the serially placed envelope to decide what abutments the patient would receive.

### Blinding

The post-clinical and radiographic parameters were assessed by two different calibrated evaluators who were also blinded for the study.

### Inclusion/exclusion criteria

All the patients recruited were more than 18 years of age with edentulous site having a minimum bone width of 6 mm and minimum bone height of 16 mm. The full-mouth plaque and bleeding scores after initial therapy were ≤ 20%. All the patients were systemically healthy with stable occlusion and a healthy periodontium.

Patients excluded were (1) those with periodontal disease; (2) smokers; (3) pregnant and lactating women; (4) patients with systemic disorders; (5) patients taking anti-inflammatory drugs, bisphosphonates in the past 6 months; (6) drug abuse.

### Statistical analysis

Data for 20 partially edentulous sites (10 in each group) were analyzed at baseline, 1 and 3 years post-early loading. All the data were subjected for statistical analysis using SPSS software version 22. Mean and standard deviation of all parameters were estimated. The normality was checked using Kolmogorov–Smirnov test and Shapiro–Wilk test. Mean values were evaluated between groups using Independent sample *t*-test. Mean values between different time points were evaluated using paired *t*-test and *P* value ≤ 0.05 was considered to be statistically significant.

### Interventions

Under topical anesthesia using 2% lignocaine with 1:80,000 adrenaline, mid-crestal incisions were made on the edentulous ridge and sulcular incisions were made on the adjacent tooth, following which buccal and lingual/ palatal mucoperiosteal flaps were minimally elevated with a periosteal elevator. Under copious saline irrigation, pilot drill was performed at 650 rpm and 20 N cm. Consequent drilling was done followed by checking for parallelism using paralleling pins. Laser-microgrooved implants* (Laser-lok implants 3.8 mm × 10.5 mm, 3.3 mm × 12 mm, 4.6 mm × 10.5 mm and 4.6 mm × 12 mm) used in our study were procured from Biohorizon which were tapered internal hex laser-lok implants which has a 2 mm micromachined collar containing microchannels for enabling connective tissue and bone attachment. Platform switching with laser-microgrooved prosthetic abutments were also procured from Biohorizon contains laser-lok microchannels on the collar (1 mm collar, 3 mm collar) with 3.5 mm and 4.5 mm platforms.

The implants were driven into the osteotomy site using an implant drive using a geared handpiece at 35 rpm with 20 N cm torque. Healing abutments were placed, and flap approximation was done followed by post-surgical instructions and medications. Chlorhexidine 0.12% was prescribed twice daily for 2 weeks. Patients were also instructed, not to brush the surgical area for 1 week, to avoid mechanical disturbance to the site. Suture removal was done after 1 week.

### Loading protocol

**Fig. 3:**
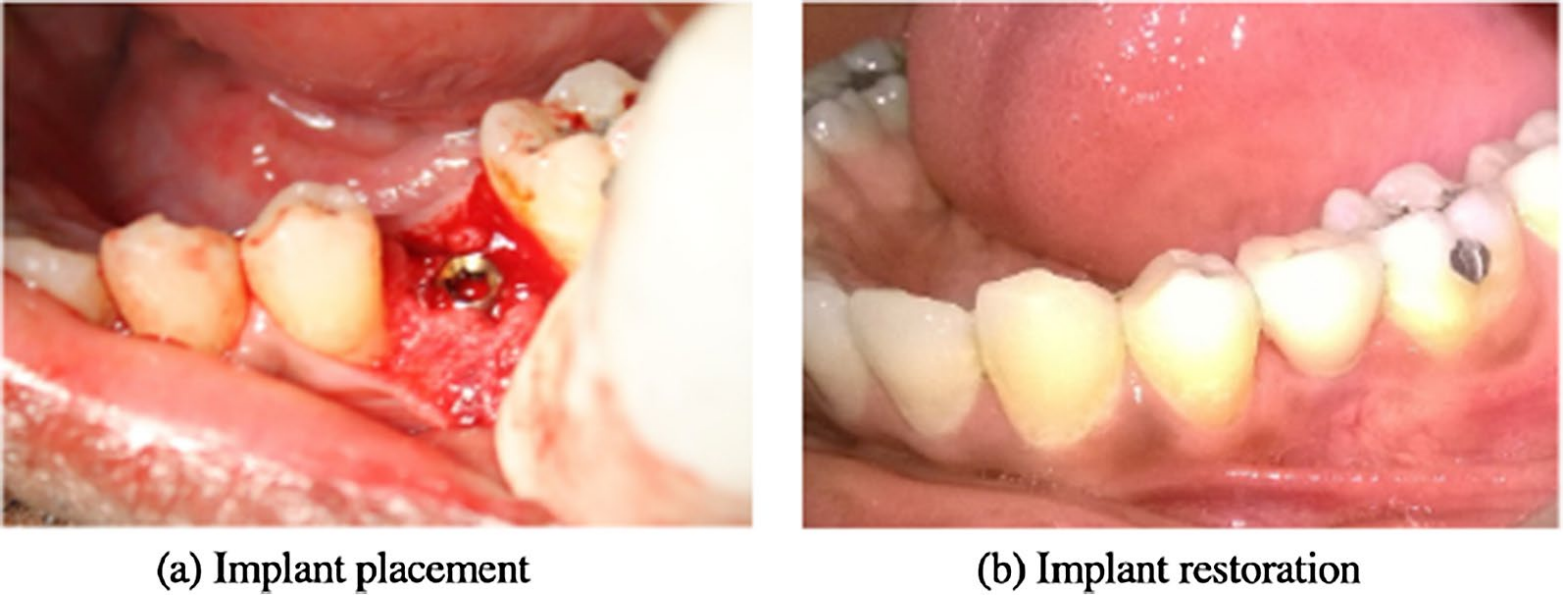
Surgical placement and restoration of implant. **a** Implant placement, **b** Implant restoration

Early loading protocol was followed wherein healing abutments were retrieved after 2 weeks of implant placement, impression was made after placing an indirect coping, followed by bite registration. The indirect coping was placed back in the impression followed by placement of milled prosthetic abutments in the patient’s mouth and final impressions were sent to the lab for preparation of metal ceramic crowns which were later cemented onto the prosthetic abutment. The metal ceramic crowns contained an entrance for the screw access of the screw retained prosthesis. Once the screw was tightened, the entrance was restored with Type II glass ionomer cement and the occlusion was reassessed for stable occlusion. Post-operatively patients were recalled on a regular basis for follow-up visits 6 weeks (baseline), 1 year, and 3 years post-early loading (Fig. 3a, b).

## Results

The study consisted of 20 patients (10 males and 10 females), 10 in the control group and 10 in the test group, recruited and treatment done between February 2013 and February 2014. The mean age estimated for the control group was 35 ± 5.8 and the test group was 36 ± 6.1, with an even gender distribution of five males and five females in both the groups. The demographic characteristics for each group are presented in Table1.

**Table 1:** Descriptive statistics of the control and the test groups

No.	Control group			Test group			*P* value
	Age (years)	Sex M/F	Tooth number	Age (years)	Sex M/F	Tooth number	
1	23	M	35	36	F	16	
2	45	F	46	43	F	47	
3	42	M	46	32	F	26	
4	32	F	46	32	M	47	
5	32	F	24	24	M	37	
6	40	F	46	47	M	36	
7	35	F	36	35	F	46	
8	38	M	36	38	M	46	
9	33	M	46	38	M	36	
10	36	M	36	41	F	36	
Mean	35 ± 5.8			36 ± 6.1			0.71

Age distribution of the patients reveals no significant differences as evident from *P* value (0.71)

No implant was lost during the follow-up period, and no sign of peri-implant inflammation or complications was observed during the follow-up.

### Full-mouth plaque score and full-mouth sulcular bleeding score

In both the control and the test groups, the full-mouth plaque scores and full-mouth bleeding scores (FMPS%, FMBS%) were initially comparable showing no statistical significance, however, at 3 years post-early loading, there was a slight rise in both the full-mouth plaque and bleeding scores (FMPS% and FMBS%) in both the groups, but still it was not statistically significant as depicted in Table 2

**Table 2:** Intergroup comparison of clinical parameters at different time points

Clinical parameters	Baseline (6 weeks post-restoration) Mean (SD)	*P* value	1 year post-restoration Mean (SD)	*P* value	3 years post-restoration Mean (SD)	*P* value
FMPS%						
Control (10)	15.3 (3.08)	= 0.812	15.4 (2.05)	= 0.687	18.3 (3.03)	= 0.88
Test (10)	15.6 (0.9)		15.5 (0.04)		17.3 (3.07	
FMBS%						
Control (10)	11.3 (4.1)	= 0.664	10.1 (0.06)	= 0.973	11.3 (1.03)	= 0.41
Test (10)	10.4 (3.7)		10.2 (1.02)		11.2 (1.08)	
SPS						
Control (10)	–	–	40%	< 0.001**	60%	< 0.001**
Test (10)	–	–	14%		14%	
SBS						
Control (10)	–	–	60%	< 0.001** –	50%	< 0.001**
Test (10)	–	–	16%		14%	
PISD (mm)						
Control (10)	3.7 (0.40)	= 0.401	3.8 (0.4)	= 0.011	4.7 (0.2)	< 0.001**
Test (10)	3.2 (0.4)		2.8 (0.1)		2.4 (0.1)	
PIAL (mm)						
Control (10)	0.00		0.00		0.00	
Test (10)	0.00		0.00		0.00	

*FMPS* full-mouth plaque score, *FMBS* full-mouth bleeding score, *SPS* site-specific plaque scores, *SBS* site-specific sulcular bleeding scores, *PISD* peri-implant sulcus depth, *PIAL* peri-implant abutment attachment loss

**Statistically highly significant

### Site-specific plaque and bleeding scores

Site-specific plaque score (SPS) taken 3 years post-early loading, revealed an escalation in the scores in control group compared to the test group with a *P* value < 0.001 which was statistically significant. Site-specific bleeding scores (SBS) taken 3 years post-early loading, also showed raised scores in the control group compared to the test group with a *P* value < 0.001, which was also statistically significant as depicted in Table 2.

### Peri-implant sulcus depth

The peri-implant sulcus depth (PISD) showed a gradual reduction in the test group (2.44 ± 0.10 mm) when compared to the control group (4.75 ± 0.20 mm) at 3 years post-early loading with a high statistical significance (*P* < 0.001). Both the control and the test group did not reveal any attachment loss as depicted in Table 2.

### Mean crestal bone loss

The mean crestal bone levels when measured at 1st year post-early loading revealed 1.08 mm in the control group and 0.76 mm in the test group, which showed a statistical significance (*P* = 0.04), whereas at 3 years post-early loading there was an evident increase in mean crestal bone loss in the control group (1.93 mm) when compared to the test group (0.61 mm) which revealed a very high statistical significance (*P* < 0.001).

**Fig. 4:**
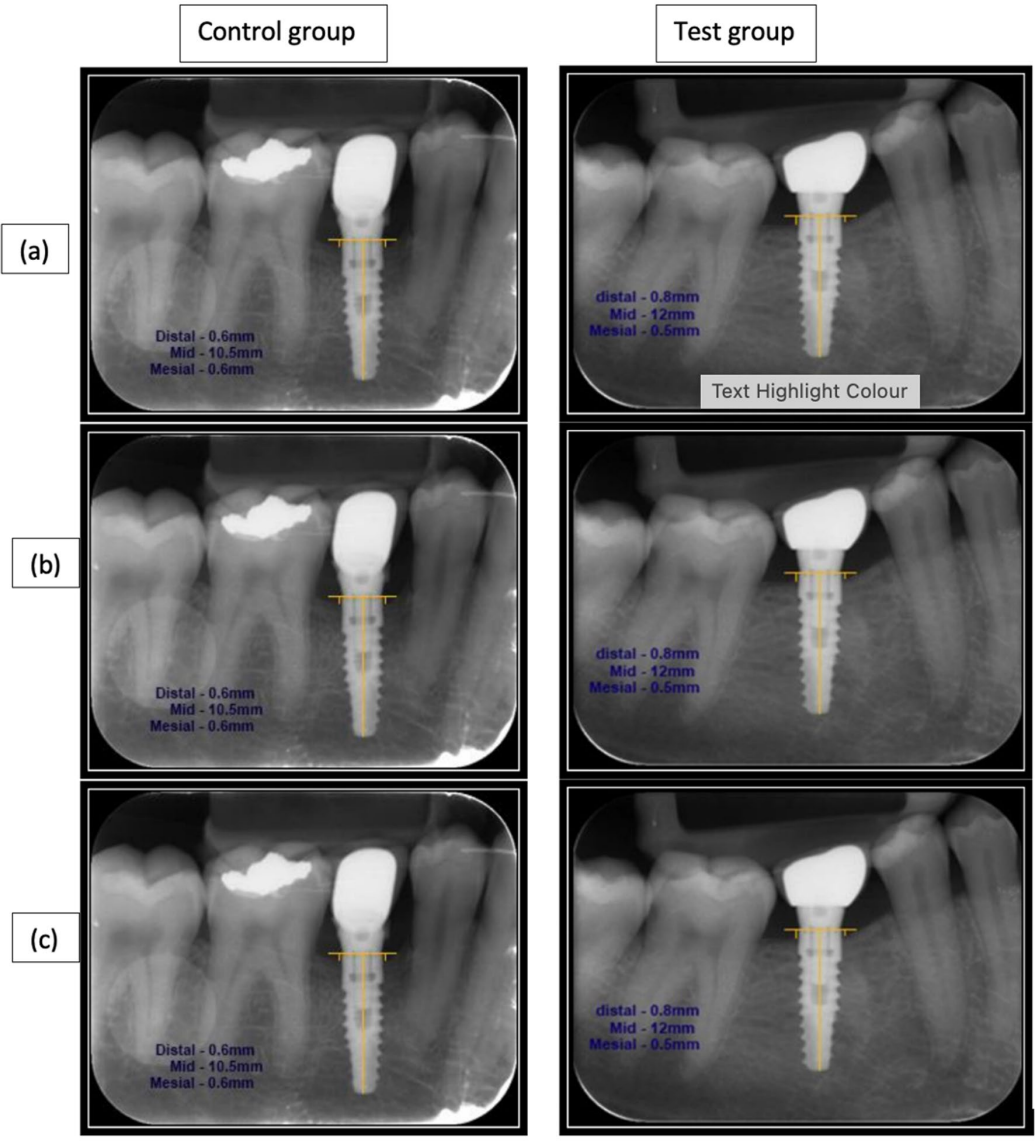
**a** Baseline **b** 1 year post-restoration **c** 3 years post-restoration

The difference in the mean crestal bone levels from the 1^st^ year (1.08 mm) of post-early loading to the 3rd year (1.93 mm) depicted an increase amount of crestal bone loss of 0.85 mm in the control group which was statistically significant with a *P* value of (*P* < 0.001) (Fig. 4a–c), whereas in the test group the radiographic changes appreciated bone gain from 0.76 mm in the 1st year of early loading to a reduction of 0.61 mm at 3rd year of post-early loading revealing a crestal bone gain of 0.15 mm with a *P* value of 0.74.

The difference in the mean crestal bone levels between the control and the test groups at 3 years of post-loading depicted − 0.85 mm of crestal bone loss when compared to + 0.15 mm of bone gain in the test group this difference revealed statistical significance with *P* value of (*P* < 0.001) (Table 3).

**Table 3:** Radiographic parameters: mean crestal bone loss in mm and the difference in mean crestal bone loss in mm at different time points

	Baseline (6 weeks post-restoration)	*P* value	1 year post-restoration (mm)	*P* value	3 years post-restoration (mm)	*P* value
MCBL (mm)						
Control (10)	NA	–	1.08		1.93	< 0.001**
Test (10)	NA		0.76		0.61	= 0.74
			< 0.04*		< 0.001**	
Difference in MCBL (mm)						
Control (10)					− 0.85	< 0.001**
Test (10)					+ 0.15	

*MCBL* mean crestal bone loss, *NA* not applicable

*Statistically significant

**Statistically significant

## Discussion

Restoration of the lost tooth using implant supported prosthesis has become a widely accepted modality of treatment in Dentistry. The general clinical assumption is that surgical trauma, and subsequent bone drilling might result in bone loss around the cervical regions of the implant leading to “saucerization” beginning from the neck of the implant and later extending apically to the first thread and so on [21]. With the significant success rates to the osseo-integrated implants, Clinicians are now focussed on options that would produce optimal aesthetic and functional results and therefore left to ponder if this observed post-restorative dieback of bone could be conquered, to enable success that would be compounded by functional and aesthetic values.

As a matter of fact, bacterial invasion at the trans-mucosal region is one of the most critical problems associated with long-term maintenance of the crestal bone levels [3]. Concerns of a possible bacterial colonization of the implant–abutment micro gap within the biological width has led clinicians to work on micro and macro design changes which could promote a soft tissue interface on the abutment, thus minimizing crestal bone loss [22].

In this study the FMPS% and FMBS% scores taken at baseline, 1 year and 3 years post-early loading had no statistical significant difference (*P* value < 0.88 and < 0.41, respectively) between the control and the test group which correlates to the fact that the patients in both the groups, were well motivated and they maintained appropriate plaque control measures throughout the study.

Ericsson et al. [23] in his study quantified two types of inflammatory response in the peri-implant tissue. One is plaque associated inflammatory cell infiltrate which was associated with the gingival sulcus, the second was abutment associated inflammatory infiltrate which was associated with the implant–abutment junction. This indicated that, like the natural tooth once the biological seal is established to the implant, it provides a protective function to isolate the crestal bone from the oral environment.

Berglundh and Lindhe [2] in their study reported that in the absence of a minimum soft tissue thickness of 3 mm, the body attempts to re-establish this dimension. The loss of crestal bone height is thus suggested to be a biologic response to create space for circum-implant fiber orientation to the implant surface thus depicting that the orientation of the fibers are very important in maintaining plaque control thus reducing the crestal bone loss.

In concordance to the studies by Ericcson et al. and Berglundh et al. [3, 22], site-specific plaque and bleeding scores taken at 1 year and 3 years post-early loading, revealed a highly statistically significant reduction in the test group (< 0.001 and < 0.001, respectively), which could be correlated to the reduction in the inflammatory infiltrate due to the soft tissue seal formed at the laser-microgrooved surface of the laser-microgrooved abutments.

The peri-implant sulcus depth taken at baseline, 1 year and 3 years post-early loading, showed a statistically significant difference between the control and test group, at both 1 and 3 years post-early loading compared to the baseline (< 0.011, < 0.001, < 0.401, respectively). In the test group there was relatively less peri-implant sulcus depth than the control at both 1 and 3 years post-early loading, which could be extrapolated to the study by Nevins et al. [13] who did a proof of principle study with laser-microgrooved implants (Biohorizon) which revealed that the laser-microgrooves were fully covered with functionally oriented collagen fibers which prevented the apical migration of epithelium, in contrast to the machined abutment surface where the oriented collagen fibers did not extend to or attach to the metal surface. Nevins et al. [14] in a canine model study revealed that the healing pattern around micro-grooved abutments demonstrated connective tissue fiber attachment oriented perpendicular to the laser-microgrooved abutments thus validating the reduction of peri-implant sulcus depth in the test group in our study.

Propelling onto the other objective of the current study which was to evaluate the role of micro-grooved implants–abutments to prevent crestal bone loss, the mean crestal bone loss was estimated to be 1.08 and 0.76 mm in the Test group at 1 year post-early loading, which was statistically significant with a *P* value of < 0.04. At 3 years of post-early loading, the crestal bone loss varied from 1.08 to 1.93 mm in the control group and 0.76 mm to 0.61 mm in the test group which was statistically significant with a *P* value of < 0.001.

The difference in the crestal bone loss from 1 to 3 years of post-early loading in the control group was 0.85 mm, whereas there was “bone gain” of 0.15 mm in the test group. This observation points out to the fact that the crestal bone dimensions varied considerably at 1 year and 3 years of post-early loading, indicating that a combination of laser-microgrooved implants–abutments indicated the ability to prevent crestal bone loss which could be in line with a recent study by Barros et al. [24] which demonstrated that laser-microgrooved abutments positively and significantly affect establishment of soft tissues with functionally oriented collagen fibers due to better levels of bone height obtained around immediate placed and loaded implants.

The greater crestal bone loss observed in the control group could be attributed to the fact that the machined abutments did not have gingival fibers oriented perpendicular to the abutment. In another study reported recently, it is understood that an inadequate epithelial attachment to the machined trans-mucosal segment could have probably caused a micro gap to be inoculated by bacteria from the peri-implant sulcus resulting in an increase in the plaque induced inflammatory state [25] thus leading to greater crestal bone loss when compared to the test group. This could be correlated to the study by Nevins et al. [15] which stated that the oriented gingival fibers that had developed in the laser-microgrooves of the healing abutment reattached to the micro-grooves on the definitive abutments, which presented no crestal bone loss compared to that of machined abutments.

Laser-microgrooved implants (Laser-lok implants 3.8 mm × 10.5 mm, 3.3 mm × 12 mm, 4.6 mm × 10.5 mm and 4.6mmX12mm) used in our study were procured from Biohorizon which were tapered internal hex laser-lok implants which has a 2-mm micromachined collar containing microchannels for enabling connective tissue and bone attachment. Platform switching with laser-microgrooved prosthetic abutments were also procured from Biohorizon contains laser-lok microchannels on the collar (1 mm collar, 3 mm collar) with 3.5-mm and 4.5-mm platforms.

Thus based on the previous published studies [15], and the results obtained from our study, we could hypothesize that laser-microgrooved implants and abutments could have probably secured a functional orientation of collagen fibers which prevented the apical migration of the epithelium, thus validating the reduced peri-implant sulcus depth and the reduced crestal bone loss in the test group.

## Summary and conclusion

The present study addresses the issue of mean crestal bone loss as a potential threat for peri-implantitis besides overcoming the same through technical modifications on the implant surface like laser-micro grooving of Implants. Within the limitations of the study, the following facts could be established, (1) The significant reduction in the Peri-implant Sulcus Depth in the Test group with a *P* value < 0.001 at 3 years post-early loading could be hypothesized based on the previous histological studies done by Nevins et al. 2010 [15] of the perpendicular orientation of fibers at the trans-mucosal segment of the laser-microgrooved abutments. (2) The significant reduction in the mean crestal bone loss in the test group with a *P* value of < 0.001 at 3 years post-early loading.

The study further emphasizes that laser-microgrooved implants, along with laser-microgrooved abutments, could be a two pronged strategy to prevent mean crestal bone loss thus promoting a healthy implant abutment junction which is free of inflammatory infiltrates, which requires further longitudinal studies for establishing the same with more clinical parameters.

## Data Availability

The data used in our study could be made available from the corresponding author only on reasonable request as it is a completely self-funded study.

## Abbreviations

FMPS: Full-mouth plaque score
FMBS: Full-mouth bleeding score
SPS: Site-specific plaque scores
SBS: Site-specific sulcular bleeding scores
PISD: Peri-implant sulcus depth
PIAL: Peri-implant abutment attachment loss
MCBL: Mean crestal bone loss
